# Semantic Verbal Fluency in Youth with Down Syndrome: Analysis of Conventional and Contextual Cluster Formation

**DOI:** 10.3390/brainsci12010009

**Published:** 2021-12-23

**Authors:** Emily K. Schworer, Shequanna Belizaire, Emily K. Hoffman, Anna J. Esbensen

**Affiliations:** 1 Division of Developmental and Behavioral Pediatrics, Cincinnati Children’s Hospital Medical Center, Cincinnati, OH 45229, USA; shequannabelizaire@gmail.com (S.B.); Emily.Hoffman1@cchmc.org (E.K.H.); Anna.Esbensen@cchmc.org (A.J.E.); 2 Department of Pediatrics, University of Cincinnati College of Medicine, Cincinnati, OH 45267, USA

**Keywords:** Down syndrome, trisomy 21, verbal fluency, psychometrics, language, child

## Abstract

Expressive language delays and executive functioning challenges are common in youth with Down syndrome (DS). Verbal fluency is one method to investigate these constructs. We examined semantic verbal fluency responses to determine patterns in response generation and the psychometric properties of coded cluster formations. Participants were 97 children and adolescents with DS ranging in age from 6 to 19 years old. The semantic verbal fluency task was administered at two time points, two weeks apart. Heterogeneity in performance was observed for responses when coded either with conventional or contextual classifications. Overall, the number of switches in conventional classifications was greater than contextual classifications. This implies that participants did not use traditional (conventional) categories to organize their semantic verbal fluency responses, but may have been using contextual strategies. However, the number of switches and cluster size variables had poor to moderate test–retest reliability, which indicated that participants did not stay consistent with their performance over the two-week testing interval, regardless of the strategies used. Therefore, conventional and contextual clusters and switches as a measure of executive control may not be appropriate for all individuals with DS and additional attention is warranted to determine the utility of response coding in this population.

## 1. Introduction

Down syndrome (DS) is associated with relative challenges with expressive language and executive function [1,2,3,4]. Within the executive function domain, working memory and cognitive flexibility are areas of pronounced difficulty in youth with DS compared to typically developing counterparts [4,5,6,7]. Within the expressive language domain, phonology, vocabulary, grammar, and pragmatics are areas of challenge for youth with DS compared to peers with typical development and intellectual disability [3,8,9,10,11,12]. Because of these challenges, both expressive language and executive function have been selected as treatment targets in the development of evidence-based behavioral and pharmacological interventions in DS. Yet, treatment studies to date have produced limited significant clinical findings, which may be in part due to measurement limitations [13,14]. Without appropriate outcome measures for youth with DS, performance and potential change cannot be properly assessed [15]. Therefore, psychometric evaluations of expressive language and executive function assessments in DS are essential to inform and support advances in future clinical trials.

### 1.1. Verbal Fluency

One method of investigating expressive language and executive function is through measurement of verbal fluency, a cognitive skill requiring language production and tracking responses with executive control [6,16,17,18]. Verbal fluency tasks assess language production by requiring the participant to generate related words in succession in response to an examiner prompt. Using verbal fluency tasks, three key components of executive control can also be distinguished [16]. First, monitoring and updating mental representations (e.g., using working memory to track responses). Next, shifting or the ability to flexibly switch between tasks or mental sets (e.g., changing to a new category once memory of one category of responses is exhausted). Lastly, inhibition of dominant responses (e.g., not responding with a favorite animal repeatedly). By assessing both expressive language and executive function, verbal fluency tasks assess domains identified as key challenges for children with DS. 

Generation of correct responses on verbal fluency tasks increases with age and receptive language level in children with DS and their typically developing counterparts [19,20]. In comparison to typically developing children matched on receptive language or mental age, children with DS produce fewer responses [6,19,21] and have a smaller number of clusters (i.e., groupings of related words) on the verbal fluency task, suggesting both the expressive language and executive function components of verbal fluency are difficult for youth with DS [19]. 

### 1.2. Verbal Fluency Assessment

There are two components of verbal fluency tasks: semantic verbal fluency and phonemic verbal fluency. Semantic verbal fluency involves generating words within a specific category, such as naming as many animals as possible. Phonemic verbal fluency involves generating as many words as possible starting with a letter, such as S [21,22] or B [19,23]. Semantic and phonemic verbal fluency tasks are used frequently within both clinical and research settings and in neuropsychological assessment to support ADHD diagnosis [24,25] as well as cognitive impairment within neurodegenerative diseases [26]. Additionally, verbal fluency has recently been recommended for use as an outcome measure in clinical trials focused on improving learning and memory in adults with DS [27]. 

The psychometrics of the semantic and phonemic verbal fluency tasks have been evaluated in previous studies and adequate test–retest reliability has been established in typically developing school-aged populations [22]. Within DS, evidence suggests the phonemic verbal fluency task is not feasible and has poor psychometrics [23]. Therefore, the phonemic verbal fluency task is not considered appropriate for most children with DS; however, findings support the use of the semantic verbal fluency task in DS [23,27]. The total number of correct responses on semantic verbal fluency tasks are shown to have adequate feasibility, test–retest reliability, and negligible practice effects, especially in children with DS 10 years and older [23,27]. Thus, semantic verbal fluency tasks have been recommended for use in clinical trials involving children and adults with DS ages 10–30 years [23,27]. Following this suggestion, hereafter, when we refer to verbal fluency, we are referring to semantic verbal fluency. 

### 1.3. Patterns in Verbal Fluency Performance

Children employ different strategies when generating responses on verbal fluency assessments [18,20,28,29]. The strategy may be conscious or unconscious and regardless, responses can be examined by grouping related words together into clusters to determine verbal fluency response patterns. Response patterns can be classified by *cluster size*; the average size of similar groups of related words [20,28]. One approach for completing the task is for participants to make clusters based on memory searches in particular categories (e.g., insects). Participants also might move on to mentally search for another category (e.g., birds) which requires the ability to alter the search criteria and switch from one category to the next. Thus, response patterns can also be classified by *switches*; the frequency that responses change to new clusters [20,28]. Participants also may not follow a strategy and instead, responses may seem random. Although there are no norms available to interpret optimal responses that correspond with executive function abilities on other assessments, both cluster size and switches are important to consider in interpretation of executive control for the verbal fluency task. A large average cluster size indicates the participant was highly productive and had a lower number of switches, whereas a small cluster size indicates there were few responses before making a switch, or there were few responses overall. Many switches may mean the participant was moving on when memory was exhausted or was unable to inhibit responses that would lead to a switch in category. Although it is not suggested in task instructions that individuals should use a strategy, in order to have a higher number of correct responses and limit repetitions, a response strategy is beneficial.

Response clusters can be categorized using *conventional* or *contextual* categories. Clusters organized conventionally use traditional categories. When grouping animals, conventional categories organize responses based upon animal species or geographic regions, as proposed by Troyer (2000) [20]. Conventional food classifications are organized based upon food groups, such as fruits, grains, and dairy foods. Clusters organized contextually use the setting or context to create categories. For animal groupings, the environment animals are found in is applied (e.g., pets or farm animals). Food groupings are completed based on the meal when foods are typically eaten (e.g., breakfast or snack foods). In previous studies, typically developing children from 4 to 7 years old primarily produced responses that were identified as contextual clustering, while older children produced responses with more conventional subcategories [30,31]. Generally, coding of response patterns has allowed researchers to observe children’s responses using both conventional and contextual coding, with clusters determined by investigators’ assessment of surrounding words to define the type of strategy being used [20]. This coding does not disentangle the likelihood of the child using one or the other forms of categorization. 

Children with DS respond with similar-sized clusters to children with typical development matched on receptive language; however, children with DS produce fewer responses overall and generate fewer switches compared to matched peers using investigator-initiated coding decisions [19]. Similar to verbal fluency coding in typically developing children, it is difficult to infer from prior literature in DS if children are scored differently due to cluster strategy. Further investigation into the psychometric properties of the format of response categories, response clusters, and switches is warranted within DS. Examining these components of verbal fluency further will provide important information about the utility of the verbal fluency task to measure language production and executive control in youth with DS, and the reliability of cluster formation scoring. 

### 1.4. Current Study

This study aimed to evaluate the verbal fluency task to determine its suitability as an outcome measure in DS. Our first aim was to describe the feasibility and overall performance on the verbal fluency task with specific attention paid to switches and cluster size coding strategies. We also compared performance based on the frequency of switches and cluster sizes using both conventional and contextual categories to understand how children with DS are generating items on verbal fluency tasks. It was hypothesized that children with DS would have larger clusters and fewer switches when responses were coded contextually, based on the similar developmental level to children who use more contextual than conventional categorization in their responses [30,31]. Children who grouped responses using contextual strategies would be expected to have larger cluster sizes and fewer switches compared to the conventionally coded classifications. Understanding the response strategies used by youth with DS will inform future coding of verbal fluency tasks and ensure coding systems are designed to meaningfully capture responses for this population. Additionally, the psychometrics of the coded switches and clusters were evaluated based on a priori criteria for practice effects and test–retest reliability. Relations to broader developmental domains were also investigated. The reliability of different coding schemes has implications for appropriately evaluating verbal fluency, and for interpreting clinical trial outcomes using verbal fluency cluster formation scoring to assess change in study participants. 

## 2. Materials and Methods

### 2.1. Participants

Ninety-seven youth with DS between the ages of 6 and 19 years participated in a larger longitudinal study on cognition in DS (M = 12.6, SD = 3.3). The average IQ as measured by the Stanford Binet, fifth edition was about 49.2 (SD = 5.5) and scores ranged from 47 to 76. Sex of the sample was approximately equal (53% male). The sample was predominantly White (87%) and non-Hispanic (93%). A portion of the participant data from the current study has also been reported in a manuscript focused on the psychometric properties of response productivity, intrusions, and perseverations in youth with DS [23]. 

### 2.2. Procedures

To be eligible for participation in this study, individuals were required to have a confirmed DS diagnosis and use English as a primary language. Nonverbal individuals were included in the broader study and a parent-reported approximate mental age of three was recommended to support participation in this study; however, no participants were excluded based on this study criterion alone. Study procedures were approved by the Streamlined, Multisite, Accelerated Resources for Trials (SMART) IRB platform at Cincinnati Children’s Hospital Medical Center (2018-0253, approved 23 April 2018). Participants were recruited from two sites in mid-west and western regions of the United States and informed consent was obtained prior to participation. Local DS associations and clinics distributed information on the study. Participants were seen for two study visits held over a two-week interval as a part of a larger longitudinal study on cognitive outcome measures in DS. Participants were administered the semantic verbal fluency task as part of a larger neuropsychological battery. 

### 2.3. Measures

#### 2.3.1. Verbal Fluency (NEPSY-II Word Generation)

A modified version of the NEPSY-II Word Generation task was used to assess verbal fluency [22]. The semantic task of the NEPSY-II has two trials, animal and food fluency. During task administration, participants were instructed to produce as many novel words as they could within 60 s for two trials, one trial on animal fluency, and another on food fluency. Participants were given the direction to name as many animals or foods as they could and provided two examples (cat and dog or pizza and milk). Examiners recorded responses that were later labeled as correct, perseverations, or intrusions. This task has been previously used with children and adults with DS [15,23,27]. Although Smeyne et al. (in press) recommend the task for children with DS 10 years and older, the current study investigated a different type of response coding methodology and therefore age was not restricted in the current study. Participants who completed the verbal fluency task at both visits were included in analyses.

To support the unique needs of the DS population, several scoring rules were added which deviated from standard verbal fluency scoring [22,23]. Repetitions of examples given by examiners during the trial were counted as correct, with subsequent repetitions counted as perseverations. Animal sounds were accepted as correct responses (e.g., “meow” for “cat” as long as “cat” was not already a response). Another deviation was the acceptance of proper nouns such as a response of “Nemo” when listing animals. See Smeyne et al. for a full explanation of scoring modifications for DS [23].

##### Response Coding

**Figure 1:**
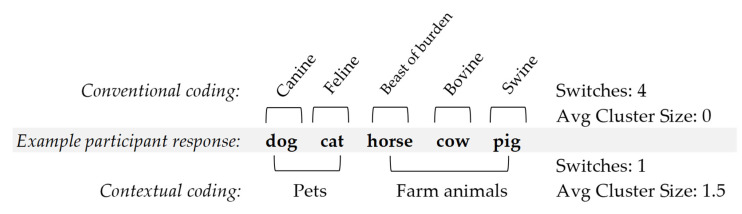
Conventional and contextual coding example. Note: As pets form a cluster of 1 and farm animals a cluster of 2, the average cluster size is 1.5.

From the two trials (animal and food), responses were organized according to two different coding schemes, *conventional* and *contextual* for each participant. Conventional clusters were composed of items grouped in traditional categories [20,28]. Contextual clusters were composed of items grouped around a context or schema. The number of words in each cluster was summed. The average cluster size was calculated by taking the sum of each cluster and dividing by the total number of clusters. Clusters were composed of a minimum of two related responses. To be considered one cluster, two responses in one category were required. Responses were then analyzed for switches. Switches were counted when participant responses shifted from one category (i.e., cluster) to another and were summed for both conventional and contextual coding methods. Thus, each participant was coded for cluster size and switches using conventional coding and again using contextual coding. This resulted in the following variables: conventional animal switches, conventional animal cluster size, conventional food switches, conventional food cluster size, contextual animal switches, contextual animal cluster size, contextual food switches, and contextual food cluster size. See Figure 1 for a coding example.

Decision rules were created to support interrater reliability. When scoring responses, examiners were encouraged to use the lowest number of switches. Twenty percent of participant data was dual coded for reliability and a kappa of 0.70 was set as the minimum criterion for reliability. Average kappa statistics were high, demonstrating strong interrater agreement for conventional animal (ICC = 0.97), contextual animal (ICC = 0.89), conventional food (ICC = 0.97), and contextual food (ICC = 0.98) categorizations. 

##### Animal Fluency

Table 1 provides the conventional and contextual classifications used for the animal fluency subtest coding. The conventional classifications were traditional categories based on Troyer (2000) and comprised of the animal species or geographic regions, such as birds or African animals [20]. The contextual classifications were items organized by context or schema, such as zoo or farm animals, and also based on previous coding schemes [20]. A participant response of “giraffe” could be categorized conventionally as an African animal and contextually as a zoo animal.

##### Food Fluency

Table 1 also lists the conventional and contextual classifications for the food fluency subtest. Conventional categories were based on food groups and individual types of food [20]. Restaurant was added as a category to account for accepting proper nouns in participant responses. The contextual classifications were comprised of the meal context during which food is typically eaten and were developed by the authors. A participant response of “taco” can be categorized conventionally as a specific meal and contextually as lunch/dinner.

#### 2.3.2. Stanford Binet Intelligence Scales, Fifth Edition (SB-5)

The SB-5 is an assessment of intelligence [32] normed using a nationally representative sample of 4800 participants ages 2 to over 85 years and commonly used to estimate cognitive abilities in studies with children and adults with DS [9,33,34]. The Abbreviated Battery IQ (ABIQ) scale consists of two routing subtests, one verbal and one nonverbal, and was administered to all participants. Traditional ABIQ scoring (M = 100, SD = 15) was used to describe the participants in the study and online deviation scoring was used for subsequent analyses [35]. Deviation scores apply a *z*-score transformation to reduce floor effects and have been used in previous studies involving children and adolescents with DS [9,34].

#### 2.3.3. Expressive Vocabulary Test, Second and Third Editions (EVT-2 and EVT-3)

The EVT-2 and EVT-3 measure expressive language and word retrieval using two forms of items focusing on either labeling or synonyms [36,37]. The examiner displays a picture and requires the child to produce the name or synonym for the picture, depending on the examiner’s prompt. This assessment is normed for children 2 years and 6 months of age through adults 90 years and older and considered appropriate for youth with DS [15]. Standard scores (M = 100, SD = 15) were used to estimate expressive vocabulary in the present study. The study switched to using the EVT-3 upon its publication which occurred after 16 participants had been enrolled with the EVT-2. Standard scores were combined across EVT-2 and EVT-3 in analyses. 

#### 2.3.4. Peabody Picture Vocabulary Test, Fourth and Fifth Editions (PPVT-4 and PPVT-5)

The PPVT-4 and PPVT-5 are measures of receptive vocabulary for children aged 2 years and 6 months to adults 90 years and older [38,39]. The measure assesses a variety of speech components including nouns, verbs, and attributes. The examiner displays four pictures and says a word. The child is required to point to or tap the picture that matches with the word spoken by the examiner. The PPVT is appropriate for use with individuals with DS [15] and standard scores (M = 100, SD = 15) were used in the current study to estimate receptive word knowledge. The study switched to using the PPVT-5 upon its publication which occurred after 16 participants had been enrolled with the PPVT-4. Standard scores were combined across PPVT-4 and PPVT-5 in analyses.

### 2.4. Analysis Plan

First, the feasibility and overall performance on the verbal fluency task were evaluated. Examiners recorded all reasons for noncompletion or uncodable data. Score distributions were examined for animal and food subtest switches and cluster sizes for both conventional and contextual classifications. The frequency of switches and average cluster size was described, and paired samples *t*-tests were used to compare performance between conventional and contextual classifications. Correlations between frequency of switches and cluster size within animal and food subtests were also investigated. Finally, the psychometrics of the coded switches and clusters were evaluated for practice effects, test–retest reliability, and associations with broader developmental domains using established standard methods for assessing these psychometric properties. Practice effects, change in scores after multiple administrations, were examined using paired samples *t*-tests and considered negligible if there were no significant differences between scores at the two time points and Cohen’s *d* effect sizes were less than 0.20. We evaluated test–retest reliability, the consistency in scores over multiple administrations, using intraclass correlations, which have set guidelines for poor (<0.50), moderate (0.50–0.74), good (0.75–0.90), or excellent (>0.90) reliability [40]. Correlations with broader developmental domains were determined by examining the relation between switches and cluster size and cognitive abilities, expressive language, receptive language, and age. Because not all data were normally distributed (Skewness < −1 or >1; Kurtosis < −2 and >2), Spearman’s rank order correlations were used.

## 3. Results

### 3.1. Overall Verbal Fluency Task Feasibility and Performance

Of the 97 children in the sample, 56 had codable data for the verbal fluency task. A portion of the data was not able to be coded because notation by examiners was not detailed enough to code switches and cluster size (animal subtest *n* = 16; 16.5% and food subtest *n* = 16; 16.5%). For the remainder of the participants without responses (*n* = 25), the reasons they were unable to complete the task were due to a lack of verbal ability (9.3%), behavioral noncompliance (2.1%), verbal refusal (1.0%), a lack of understanding the task (4.1%), only completing the task at one visit (5.2%), or no in-person visit due to COVID-19 (4.1%). For more detail on task feasibility and age-related differences in performance, see Smeyne et al., in press.

The 56 participants with codable data for the animal fluency task were between the ages of 7 and 19 years (M = 13.5, SD = 2.9) and had an average IQ of 49.3 (SD = 6.3). The 56 participants with codable data for the food fluency task were between 6 and 19 years old (M = 13.4, SD = 3.0) and had an average IQ of 49.6 (SD = 6.5). Sex of the subsamples was comparable to the overall sample (57% male for animal fluency and 55% male for food fluency). Participants had similar response productivity on animal (M = 9.11, SD = 4.92) and food (M = 10.30, SD = 4.41) subtests.

Table 2 presents minimum, maximum, and median scores. Concerns for both skewness and kurtosis were identified (see Table 2). Average frequency of switches for the conventional animal fluency task was 9.0, with an average cluster size of less than 1 (0.26) at Time 1 (see Table 3). Scores were similar for the food trial with an average of 7.6 switches between categories and an average cluster size that was slightly higher (0.46) but still less than 1. Analysis of responses using contextual classification yielded a different pattern. Animal switches had an average of 5.7 switches between categories and a corresponding average cluster size of 1.08. The average number of food switches was 4.1 and had a corresponding average cluster size of 1.89. The variance in performance for both conventional and contextual classifications was sizable (Table 3). See Table 2, Table 3 for full descriptive information on switches and average cluster sizes.

Performance was compared across conventional and contextual classification methods. For the animal subtest, there were significantly more switches using conventional coding compared to contextual coding, *t*(55) = 9.99, *p* < 0.001, *d* = 0.74. Correspondingly, average cluster size was larger using contextual coding, *t*(55) = −7.62, *p* < 0.001, *d* = 1.38. Results were similar for the food subtest. There were significantly more switches using conventional coding compared to contextual coding, *t*(55) = 11.80, *p* < 0.001, *d* = 1.19, and the average cluster size was larger using contextual coding, *t*(55) = −7.48, *p* < 0.001, *d* = 1.37. See Table 3 for means and standard deviations.

Finally, the relations between the number of switches and average cluster sizes were investigated using Spearman’s rank order correlations. Significant correlations were observed between switches and average cluster sizes for the conventional classification food subtest (ρ = −0.28, *p* < 0.05) and contextual classification animal (ρ = −0.48, *p* < 0.001) and food (ρ = −0.71, *p* < 0.001) subtests. Participants with a small number of switches had a larger average cluster size than those with a greater number of switches. There was no significant correlation between the number of switches and the average cluster sizes for the conventional classification animal subtest (ρ = 0.12, *p* = 0.39).

### 3.2. Psychometric Properties of Coding Metrics

Practice effects were examined over the two-week testing interval. Overall, significant practice effects were not identified between trials at time one and time two, indicating there were no group-level improvements in performance at the second visit (Table 3). Test–retest reliability of the switches and cluster sizes was poor to moderate, regardless of conventional or contextual classifications and animal or food subtest (Table 3). Psychometric properties were also examined for a restricted age range (10 and older), but differences in indices were not evident [23].

Verbal fluency switches were associated with some of the broader developmental domains (cognitive abilities, expressive language, receptive language, and age; see Table 3). First, conventional animal cluster size and contextual animal cluster size were associated with cognitive abilities. Conventional animal cluster size, conventional food switches, and contextual animal cluster size were related to expressive language abilities. Conventional animal cluster size and contextual animal cluster size were correlated with receptive language skills. Finally, contextual food switches were associated with participant age (Table 3).

## 4. Discussion

This study examined verbal fluency responses in youth with DS to determine patterns in response generation and the psychometrics of cluster formations. Heterogeneity was observed for the total number of response clusters/switches among clusters and average cluster size. There were significant differences based on coding classifications (i.e., conventional vs. contextual). For the psychometric evaluation, there were mixed results. First, there were no significant practice effects between time one and time two, indicating that there was little improvement to performance from the previous administration of the task. However, test–retest reliability was poor to moderate, with mixed associations with broader developmental domains. Thus, conventional and contextual clusters and switches as a measure of executive control may have limited clinical utility for individuals with DS (ages 6–19 years).

### 4.1. Cluster Size and Frequency of Switches

Heterogeneity in performance was observed for average cluster sizes and the frequency of switches. Although the range in performance for the frequency of switches was similar between conventional and contextual categorization, on average there were more switches, and thus more clusters when scored with conventional compared to contextual categorizations. The range in performance was different between coding types when comparing cluster size. Average cluster size of the conventional classifications had a more restricted range than contextual classifications and overall cluster size was larger with the use of contextual coding. This implies that contextual or schematic strategies may have been employed by youth with DS and participants did not use animal species or geographic regions to organize their verbal fluency responses. The tendency for contextual categorizations to be used by children with DS was in line with study hypotheses and replicates previous research that found a tendency for contextual groupings in cluster formation in similarly aged children with DS [19]. It also corresponds with studies on typically developing children of a similar mental age, in which contextual strategies were used over conventional classifications in verbal responses [30,31].

There were associations identified between the number of switches and average cluster size within subtests. The relation between the number of switches and cluster size could mean that those using more executive control with their responses would have larger cluster sizes and infrequent switches, which were observed when coding contextually. For example, if a participant was able to name farm animals, then switch and begin naming aquatic animals, they would have larger cluster sizes than they would if switching back and forth between categories. Smaller cluster sizes corresponding with more frequent switches would be an indicator of little executive control, which was observed when coding conventionally, but as stated earlier it may also mean participants were not grouping based on conventional categories.

### 4.2. Clustering and Switching Psychometrics

Although practice effects were negligible for cluster and switching verbal fluency scoring, problems were apparent with the poor to moderate test–retest reliability indices. These findings suggest that although improvements were not made between the two testing visits, participants did not stay consistent with their performance over the two-week testing interval. This lack of consistency in scores is a challenge for practical use of the coding system in longitudinal studies or clinical trials. Despite clear patterns in contextual over conventional classifications, without consistency in responses, the utility of the tool to assess executive control through examination of cluster formations is limited. Additionally, restricting the chronological age to 10 and up as suggested by Smeyne et al. (in press) did not improve psychometric properties (data available from the first author). Although there was a lack of consistency in scores for switches and cluster size, the total number of correct semantic verbal fluency responses were shown to have moderate test–retest and negligible practice effects in youth with DS, supporting the utility of the task as an outcome measure more broadly [23].

There was some evidence of associations between cluster size and frequency of switches with broader developmental domains. First, the cluster sizes were correlated with expressive language for the animal subtest. Conventional food switches were also correlated with expressive language. Taken together, these findings might represent an emphasis on expressive language abilities in scores intended to assess executive components of the verbal fluency task. However, the verbal nature of the fluency task and the moderate strength in correlations suggest there may be only a modest expressive language confound in cluster and switching scoring.

Additionally, a moderate correlation was identified between animal cluster size and receptive vocabulary in both conventional and contextual classifications. This result was not in line with previous work, which reported no significant relation between cluster sizes with receptive vocabulary in DS [19]. It may be the case that the larger sample size in the current study attributed to a more accurate assessment of this association or that the differences in coding schemes between studies impacted the relation between variables.

There were also associations with cognitive abilities and conventional animal cluster size and contextual animal cluster size. This result provides evidence that cognitive abilities corresponded with cluster formation and support the theory that memory and other executive processes that overlap with overall cognition are tapped in the animal verbal fluency subtest. The lack of correlations found between the food subtest and broader developmental domains warrants further evaluation to support interpretation. It is possible that food cluster formation abilities do not correspond with cognitive abilities.

Finally, one correlation with age was identified in the current study. Specifically, older participants had a lower number of contextual food switches. One interpretation of this modest correlation is that older children with DS were grouping more words in each category together and thus switched between categories of responses less frequently. However, given the lack of association between age with switches in conventional food or with conventional or contextual animals, it is also possible that the association between age and contextual food switches is spurious. Confirmation of findings is warranted. The lack of findings between age and many of the verbal fluency clusters and switches in DS corresponds with previous research that found no association between age and the total number of correct responses on the verbal fluency task in children and adolescents with DS [23].

### 4.3. Study Limitations and Future Directions

Our study was limited in that there was only a two-week interval between testing periods. In the future, it may be helpful to evaluate longer periods between testing in order to match use in clinical trials in DS and determine if there are changes in performance over these longer periods. Limitations with the coding system are also apparent. For example, the coding system, especially in the contextual categorization may be culturally dependent. This is particularly true in the food category. Previous studies have included categories such as fish’n’chips [19] or curries [29] from studies based in the UK and India, respectively. Cultural differences in food types make it challenging for establishing internationally appropriate coding. There was also limited diversity in the sample which restricts the generalizability of the study. More work is also needed to determine the ecological validity of verbal fluency tasks to support generalizability to daily functioning. A way this can be accomplished is by looking at verbal fluency in conjunction with everyday measures or clinical assessments of language and executive functioning and including comparisons between coded responses using an updated coding system and executive function.

## 5. Conclusions

This study provides preliminary evidence that children with DS may be organizing verbal fluency responses contextually rather than conventionally, as they appear to be engaging in fewer switches and larger cluster sizes in contextual classification relative to conventional coding. However, not all psychometric criteria were met, indicating these measures may not be reliable ways to assess verbal fluency performance in DS. Despite the limited utility of the coding system in youth with DS, additional studies are needed to determine if the coding system is meaningful for adults with DS or if a modified version of the coding scheme would be useful for research. Productivity of verbal fluency responses (i.e., number of correct responses) remains recommended for youth older than 10 years [23], but additional research is needed to develop valid classifications of participant responses.

## Figures and Tables

**Table 1:** Animal and food classifications for conventional and contextual coding schemes adapted from Troyer (2000).

Animal Classifications		Food Classifications	
*Conventional*	*Contextual*	*Conventional*	*Contextual*
African animals	Farm animals	Beverages	**Breakfast**
American animals (north and south)	Pets	Condiment	**Lunch/Dinner**
Arctic animals	**Woodland animals**	Dairy	**Snack**
**Asian animals**	Water animals	Flavoring	**Dessert**
Australian animals	**Zoo animals**	Fruits	
Amphibian/Reptile		Grain products	
**Bat**		Meats/**Protein**	
Beast of burden		**Restaurant**	
Bird		Specific meals/Dishes	
Bovine		Sweets and snacks	
Canine		Vegetables	
Feline			
Fish/Aquatic			
Insect			
Primate			
Rodent			
**Swine**			

Note. Bold categories were added to the coding system published by Troyer (2000) [20]; deer and rabbit categories were removed as both were included in the American animals category.

**Table 2:** Performance on verbal fluency tasks at Time 1; animal subtest *n* = 56 and food subtest *n* = 56.

	Min	Max	Median	Skewness	Kurtosis
*Conventional Classification*					
Animal Switches	1	25	9	0.87	1.60
Animal Cluster Size	0	1.50	0.19	1.83	4.22
Food Switches	3	15	8	0.42	−0.51
Food Cluster Size	0	1.80	0.32	1.01	0.49
*Contextual Classification*					
Animal Switches	0	21	4.50	1.76	4.21
Animal Cluster Size	0	4	0.75	1.06	0.70
Food Switches	0	14	3	1.24	2.36
Food Cluster Size	0	8	1.29	1.68	2.93

**Table 3:** Means, practice effects, test–retest reliability, and associations with broader developmental domains for verbal fluency tasks; animal subtest *n* = 56 and food subtest *n* = 56.

	Time 1 Mean (SD)	Time 2 Mean (SD)	*t*	Cohen’s *d*	ICC	ABIQ ^a^	EVT	PPVT	Age
*Conventional Classification*									
Animal Switches	9.00 (4.80)	8.21 (4.03)	1.66	0.18	0.68	0.13	0.22	0.16	0.14
Animal Cluster Size	0.26 (0.30)	0.29 (0.31)	−0.63	0.10	0.40	0.31*	0.38 **	0.38 **	−0.03
Food Switches	7.63 (3.21)	7.52 (3.04)	0.26	0.04	0.52	0.12	0.27 *	0.17	−0.22
Food Cluster Size	0.46 (0.43)	0.44 (0.52)	0.27	0.04	0.26	0.07	0.19	0.06	0.12
*Contextual Classification*									
Animal Switches	5.68 (4.15)	5.18 (3.21)	1.06	0.13	0.54	−0.09	0.11	0.08	−0.02
Animal Cluster Size	1.08 (0.89)	1.06 (0.94)	0.15	0.02	0.35	0.45 **	0.31 *	0.30 *	0.05
Food Switches	4.11 (2.70)	3.63 (2.32)	1.42	0.19	0.49	0.11	0.19	0.21	−0.31 *
Food Cluster Size	1.89 (1.65)	2.21 (2.41)	−1.19	0.15	0.52	0.05	0.10	−0.04	0.25

* *p* < 0.05; ** *p* < 0.01; ^a^ Stanford–Binet, fifth edition deviation scores.

## Data Availability

The data that support the findings of this study are available from the corresponding author upon reasonable request.
